# Co-existence of severe coarctation of the aorta and aortic valve stenosis in adulthood: a case report

**DOI:** 10.11604/pamj.2021.39.46.27782

**Published:** 2021-05-19

**Authors:** Mahassine El Harras, Amal El Ouarradi, Salma Abdeladim, Ilham Bensahi, Sara Oualim, Fatimazahra Merzouk, Najwa Benslima, Hicham El Malki, Said Makani, Mahdi Ait Houssa, Chafik Kettani, Mohamed Sabry

**Affiliations:** 1 Department of Cardiology, Mohammed VI University of Health Sciences Cheikh Khalifa Hospital, Casablanca, Morocco,; 2 Department of Radiology and Medical Imaging, Mohammed VI University of Health Sciences Cheikh Khalifa Hospital, Casablanca, Morocco,; 3 Department of Cardiovascular Surgery, Mohammed First University, Faculty of Medicine and Pharmacy, Oujda, Morocco,; 4 Department of Cardiovascular Surgery, Mohammed VI University of Health Sciences Cheick Khalifa Hospital, Casablanca, Morocco

**Keywords:** Aortic valve pathology, aortic coarctation, bicuspid aortic valve, aortic valve stenosis, case report

## Abstract

Aortic coarctation is a congenital heart disease that usually presents and is treated in the childhood. The aortic coarctation is often associated with concomitant cardiac pathologies, such as aortic stenosis and bicuspid aortic valve. We report the case of a 56-year-old man, admitted in our cardiologic unit, for sudden onset of chest pain, dyspnea and syncope. Aortic coarctation with aortic valve stenosis was diagnosed. The aortic valve was successfully replaced in the first stage; the coarctation had to be treated in a second time.

## Introduction

Coarctation of the aorta is a congenital vascular lesion typically diagnosed and corrected in childhood or early adulthood [1]. There are a few case reports of patients first diagnosed as having coarctation of the aorta after their 50´s [2]. This congenital heart disease is often associated with other cardiac congenital diseases [3]. We report the case of a 56-years-old man who was first diagnosed as having coarctation of the aorta and aortic valve stenosis.

## Patient and observation

We report the case of a 56-years-old man, treated for hypertension since 5 years, controlled with association of valsartan/amlodipine. He has a 1 year history of intermittent dyspnea level II-III of the New York Heart Association (NYHA) classification. He was admitted to our hospital for chest pain, a short loss of consciousness with spontaneous recovery, associated with an acute dyspnea level III -IV of the NYHA classification. Clinical examination revealed diminished femoral pulses, 120/79 mmHg as blood pressure in the upper limbs and 100/60 in the lower limbs with a difference of 20mmhg. Auscultation over the aortic area revealed a harsh meso-telesystolic crescendo-decrescendo murmur 6/6 radiating to carotids, abolition of B2 and signs of congestive left heart failure. Pulses are perceived in the upper and lower extremities.

**Figure 1:**
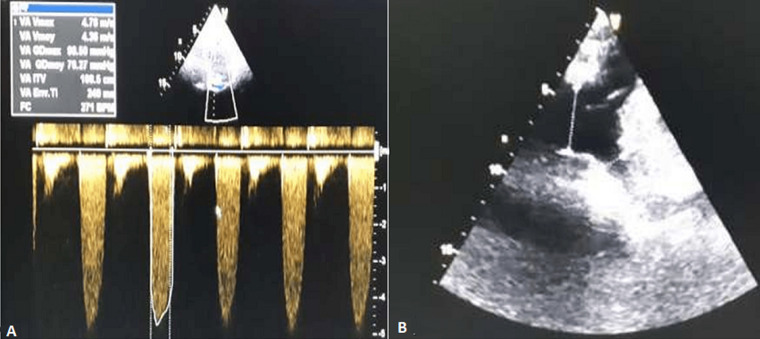
continuous aortic Doppler, A) showing the peak transvalvular pressure gradient and mean transvalvular pressure gradient respectively of 164/74 mmHg, the sub sternal incidence; B) showed an enlarged ascending aorta and subclavian artery

**Figure 2:**
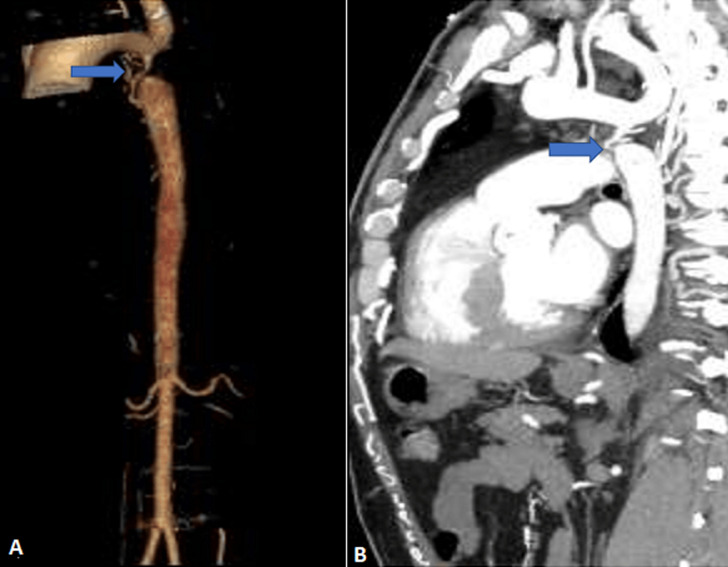
volume rendering A) and sagittal B) reconstruction of aortic CT images, showing a very tight narrowing of 2cm downstream of the origin of the supraclavicular artery (arrow)

The Electrocardiography (EKG) demonstrated a sinus rhythm 85 beats per minute, normal axis and left ventricular hypertrophy (Corenell index at 35mm). Chest radiography showed interstitial oedema with disappearance of the aortic knob and rib notching. Echocardiography revealed moderate systolic dysfunction, with an ejection fraction of only 45%. The aortic valve was totally calcified, it was impossible to determine the number of cusps, the peak transvalvular pressure gradient and mean transvalvular pressure gradient were respectively up to 164/74 mmHg, and the valve area was reduced to 0.6cm^2^ (0.32cm^2^/m^2^ of body surface area). The sub sternal incidence showed an enlarged ascending aorta and subclavian artery (Figure 1). Cardiac catheterization revealed normal coronary arteries. Computed tomography (CT) angiography was performed and showed complete occlusion of the descending aorta with extensive collateral circulation (Figure 2).

**Figure 3:**
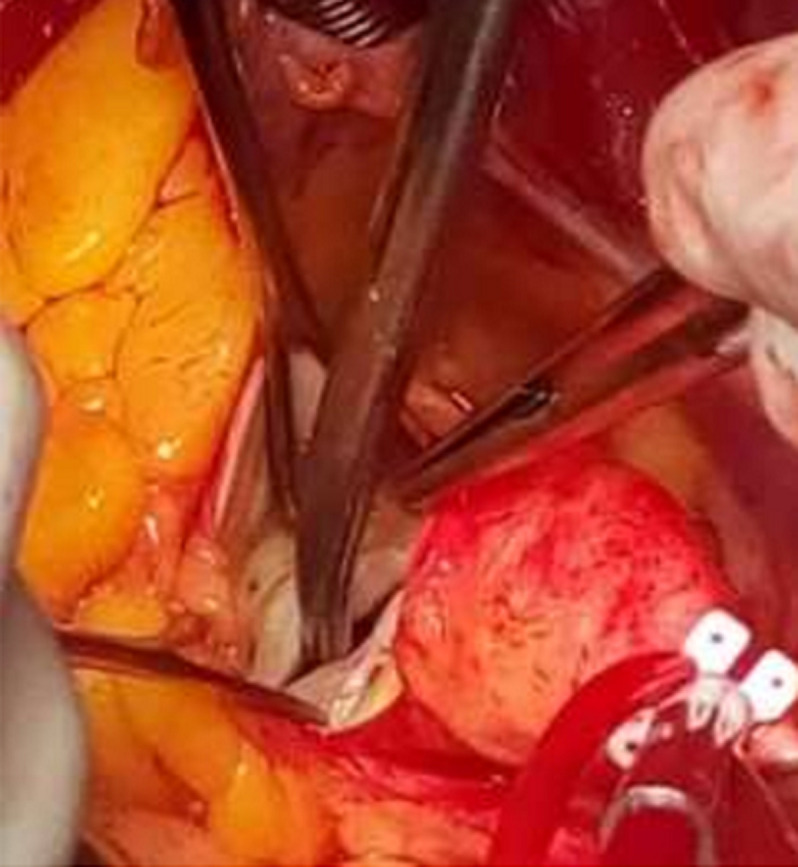
per operative view of the bicuspid aortic valve

After consulting the heart team, the decision to repair the aortic stenosis was made. The per operative analysis showed a bicuspid valve (Figure 3). The aortic valve was replaced with a bileaflet mechanical valve one-x size 21mm, both the ascending aorta and the right iliac artery were cannulated for arterial return and a single two-stage venous cannula was inserted into the right atrium. Extracorporeal circulation was started. The cross-clamping time was 86 min and the total bypass time was 120 min. The patient´s postoperative course was uneventful and he was discharged from the hospital on postoperative day 8. Surgical repair of the coarctation was planned as a second stage, but the patient refused. One month later the patient felt a clear clinical improvement, without any chest pain, syncope nor dyspnea. The echocardiographic control showed a decrease in the transvalvular gradient both peak and mean gradient (6.59/2.52mmHg).

## Discussion

Coarctation of the aorta is a congenital vascular malformation, defined as a narrowing of the thoracic aorta after the left subclavian artery. It accounts for 5-8% of all congenital heart diseases. It may be associated with other congenital heart diseases such as atrial septal defect, ventricular septal defect, patent ductus arteriosus, or aortic bicuspidia. The aortic bicuspidia is most commonly encountered with an estimate of 50 to 85%. It may be accompanied by aortic stenosis or regurgitation [1,3,4]. Rosos-Hesselink and all reported in their study that the aortic valve bicuspid was present in 62% of patients with aortic coarctation. Aortic valve disease was present in 63%, rising to 70% in patients with a bicuspid valve [5]. This congenital defect is often diagnosed during childhood. About 10% of patients with coarctation remain asymptomatic and are discovered after the age of 50 [2]. Signs and symptoms depend on the severity of coarctation and its associated congenital disease. Significant aortic coarctation may be asymptomatic in the presence of an extensive collateral circulation [6]. In the adulthood it can be manifested by symptoms such as headache, shortness of breath, abdominal angina, claudication, severe or resistant hypertension. The clinical examination may reveal blood pressure gradient between the upper and lower limbs, carotid hyper-pulsatility, a weak or absent pulse in the lower extremities. Systolic para sternal harsh heart murmur radiating to the back may be found [7]. Most unfortunately, it can be diagnosed then complication like aneurysm rupture, aortic dissection, accelerated coronary artery disease, stroke and congestive heart failure, or when associated heart malformation became symptomatic. The increased incidence of aortic valve problems associated with coarctation of the aorta is most likely due to hypertension and the significant stress caused by the coarctation gradient on the aortic valve and aortic wall. The presence of an associated bicuspid accelerates the valve degeneration and the formation of early calcifications [4,5]. Echocardiography provides information regarding site, structure, and extent of coarctation, left ventricular function and left ventricular hypertrophy and other cardiac congenital diseases [7]. Cardiovascular magnetic resonance and cardiovascular computed tomography are the preferred non-invasive techniques to evaluate the entire aorta in adults [8].

The surgical treatment of this congenital heart disease has evolved considerably. The endovascular treatment became the best and safest approach [7]. When coarctation is combined with significant aortic stenosis, it presents a surgical challenge. Different techniques have been used: either Aortic valve replacement and surgical correction of the coarctation in one single step, or staged approach. Simultaneous correction of aortic coarctation associated with aortic valve replacement can be performed either by median sternotomy only, which allows safe access for repair of the cardiac lesion and for simultaneous ascending trans pericardial aorto-degenerative bypass and descending aorto-thoracic bypass [9,10], or through a median incision in the sternum to perform aortic valve replacement and a median incision in the abdomen to perform ascending aorto-basal bypass of the abdominal aorta by diaphragm muscle grafting [11]. The advantages of the second option include easier hemostasis compared with ascending and descending aortic bypass, hemodynamic stability, less spinal cord ischemia. On the other hand, some changes in gastrointestinal function and intestinal adhesions may appear. When valve replacement is performed first as part of a staged approach, there is a potential difficulty in reestablishing the patient's circulation and significant hypoperfusion of organs distal to the coarctation may occur. If the coarctation is corrected first, the aortic valve pathology may cause hemodynamic instability [11,12]. The combined use of endovascular and interventional surgical techniques provides a significant advantage in some cases. In adult congenital heart center, coarctation with cardiac pathology can be successfully managed with a 2-stage procedure. In the first phase, the cardiac pathology is surgically corrected and coarctation managed with endovascular procedure in the second phase, with acceptable procedural risk. Intermediate and long-term results still need to be evaluated [13]. There are no guidelines on which lesion should be corrected first or on the best type of procedure. The choice is left to the heart team.

Our patient was asymptomatic apart from his high blood pressure thanks to the presence of the extensive collateral circulation. He suffered from dyspnea when his aortic valve degenerated. We opted for a two-step correction, starting with a successful aortic valve replacement; the patient refused surgical treatment of his coarctation. During the six-month follow-up, the patient was asymptomatic.

## Conclusion

The management of coarctation of the aorta associated with aortic valve disease is a big challenge. There is a need to consider whether a one-step or two-step procedure may be appropriate for these patients, depending on their individual condition, the skills of the team, and the availability of the technical platform.
